# MicroRNAs and epithelial-mesenchymal transition in prostate cancer

**DOI:** 10.18632/oncotarget.11708

**Published:** 2016-08-30

**Authors:** Kirandeep Sekhon, Nathan Bucay, Shahana Majid, Rajvir Dahiya, Sharanjot Saini

**Affiliations:** ^1^ Department of Urology, Veterans Affairs Medical Center, San Francisco and University of California San Francisco, CA, USA

**Keywords:** microRNAs, prostate cancer, EMT, metastasis, recurrence

## Abstract

Prostate cancer (PCa) is a leading cause of male cancer-related deaths. A significant fraction of prostate tumors are very aggressive, often metastasizing to bone, causing significant morbidity and mortality. Also, PCa is associated with high rates of recurrence, often attributed to the existence of cancer stem cells. Epithelial-mesenchymal transition (EMT), a process characterized by decreased expression of epithelial genes and increased expression of mesenchymal genes, plays a critical role in tumor invasion, metastasis and recurrence. In PCa, EMT has been implicated particularly in the context of metastatic disease and microRNAs have emerged as critical post-transcriptional regulators of PCa EMT. In this review, we summarize the role of miRNAs in PCa EMT that play a role in progression, metastasis and recurrence. Studies till date suggest that microRNAs mediate efficient and reversible control of PCa EMT via multiple mechanisms including either by (i) directly repressing single or multiple EMT-TFs or regulating cytoskeletal components (epithelial/mesenchymal genes) or (ii) regulating key signaling pathways involved in EMT. Oncogenic microRNAs often act as EMT promoters by repressing epithelial characteristics and tumor suppressive miRNAs act by inhibiting mesenchymal progression. Further, EMT is mechanistically linked to stem cell signatures in PCa and several miRNAs implicated in EMT have been reported to influence PCa stem cells. Loss of EMT-inhibiting miRNAs and/or gain of EMT promoting miRNAs lead to induction of PCa EMT, leading to tumor progression, metastasis and recurrence. Restoring expression of tumor suppressive miRNAs and inhibiting oncogenic miRNAs represent potential therapeutic opportunities to prevent disease metastasis and recurrence.

## INTRODUCTION

Prostate cancer (PCa) is the most commonly diagnosed cancer among men [1, 2]. It is the second leading cause of male cancer-related deaths in US with an estimated 26,120 deaths in 2016 [3]. Prostate tumors are often indolent [4] but a significant fraction of tumors are very aggressive, often metastasizing to bone and other organs, causing significant morbidity and mortality [5]. PSA (prostate-specific antigen) screening has revolutionized the clinical management of prostate cancer [6]. However, owing to the drawbacks of PSA including low specificity and inability to differentiate between indolent and aggressive prostate tumors, prostate cancer clinical management is often challenging. Treatment options for advanced metastatic disease are limited and cause marginal increases in survival [7, 8] Also, PCa is associated with high rates of disease recurrence, with ~40% of localized PCa cases having relapse after initial therapy [9] and tumor progression to a hormone refractory/castration resistant stage [1, 10] that is essentially untreatable [1, 11]. A major challenge is the elucidation of underlying molecular pathways of PCa progression, recurrence and metastasis that will open new avenues for the design of effective diagnostic, prognostic and therapeutic strategies for better clinical management of the disease.

Epithelial cancer cells convert to motile, mesenchymal cells by undergoing an epithelial-mesenchymal transition (EMT) [12–14] that plays a critical role in cancer metastasis and tumor invasion. EMT is characterized by changes in gene expression profile, including decreased expression of epithelial genes, such as E-cadherin (also known as cadherin 1 [CDH1], and increased expression of mesenchymal genes, such as N-cadherin and vimentin. The EMT process is a complex genetic program that involves complex interactions of diverse inducers [e.g. Transforming Growth Factor β (TGFβ), Fibroblast Growth Factor (FGF), multiple signaling pathways (e.g. Wnt signaling) and is coordinated in a large part by several EMT-transcription factors (TF). EMT and its reverse process, mesenchymal-epithelial transition (MET), play a critical role during organogenesis and tissue differentiation during normal embryonic development [14–17]. These embryonic processes are reactivated by primary tumor cells to acquire motility, invasiveness and form secondary tumors at distant sites. Following EMT, cells detach from the primary tumor, circulate through blood or lymphatic routes, and colonize a distant site wherein the cells undergo MET to revert back to their immobile epithelial states, leading to development of secondary tumors after metastasis [16]. It has been proven that the loss of the epithelial marker, E-cadherin (E-cad/CDH1), is a causal factor in cancer progression [18]. EMT promotes stem cell properties endowing cancer cells with the capabilities of continued self-renewal [19].

Several EMT-transcription factors (EMT-TFs) repress the expression of E-cad and other epithelial markers and induce the expression of mesenchymal markers [16]. There are three major families of transcription factors that control EMT - Zeb (Zeb1/Zeb2); zinc finger Snail (Snail/Slug); and basic helix-loop-helix families (e.g. Twist1) [16]. The Snail and Zeb family of transcription factors are highly conserved zinc finger transcription repressors that bind to E-box like promoter elements in DNA, thereby influencing gene expression and tumorigenesis. Zeb1 and Zeb2 have been shown to regulate the expression of various EMT- and tumor related genes and thereby have been implicated in EMT, tumorigenesis and metastasis [20–24]. It is now well established that EMT-TFs play important roles in tumorigenesis. Snai1, Snai2, Zeb1, Zeb2 and Twist expression have been correlated with increased metastasis and poor prognosis in human tumors [25]. In addition, epigenetic mechanisms also play a role in EMT and epigenetic regulators like Bmi1 promote EMT [26]. Several signaling pathways regulate EMT and include key molecules such as TGFβ (transforming growth factor beta), fibroblast growth factor receptors (FGFRs) and platelet-derived growth factor (PDGF) [18]. TGFβ is a well-known inducer of EMT [15, 16, 18] that works by targeting the EMT-TFs through Smad-dependent and -independent transcriptional pathways [15]. Other soluble growth factors that have been shown to induce EMT include EGF, IGF1, and β2-microglobulin [15]. In addition, TGFβ cross-talks with signaling pathways such as Wnt, Notch, NF-κB and Receptor Tyrosine Kinases (RTKs) to induce EMT. Signaling via RTKs and their downstream effectors such as PI3K, MAPK is crucial to regulate EMT [18].

MicroRNAs recently emerged as important regulators of EMT and MET in various cancers [16, 18]. MicroRNAs (miRNAs) are small, noncoding RNAs that suppress gene expression post transcriptionally via sequence-specific interactions with the 3′- untranslated regions (UTRs) of cognate mRNA targets [27]. miRNAs suppresses their cognate target genes through the destabilization or cleavage of the mRNA and/or translational inhibition [14, 16]. A single miRNA can target many mRNAs, and conversely, many miRNAs can target a single mRNA [16]. MicroRNAs regulate EMT through their ability to post-transcriptionally regulate various components of the process such as EMT-TFs, epithelial and mesenchymal genes or by regulating key signaling pathways [14, 16, 18]. A role for miRNAs that affects EMT and progression and metastasis of human cancers is increasingly emerging [25, 28, 29]. In prostate cancer, the role of miRNAs in EMT is being explored and there are few reports. Here we review and summarize the role of miRNAs in prostate cancer EMT that play a role in prostate cancer progression, metastasis and tumor recurrence.

## PROMINENT MICRORNAS REGULATING EMT IN VARIOUS CANCERS

miRNAs have been estimated to target over one-third of the human genome [14, 18]. In view of the enormous regulatory potential of miRNAs, deregulated miRNA expression has been revealed in various disease states, including cancer [30, 31]. miRNAs can be oncogenic or tumor-suppressive [32]. In addition, several miRNAs called ‘metastamirs’ influence tumor metastasis [14]. miRNAs affect EMT and MET and thereby regulate progression and metastasis of various human cancers [25, 28, 29]. Prominent examples are the miR-200 family and miR-205 that regulates EMT through direct targeting of Zeb1 and Zeb2 [25, 33, 34]. miR-9, MYC/MYCN-induced miRNA, directly targets E-cadherin and breast cancer metastasis [35]. miR-103/107 induces EMT by downregulating miR-200 levels, and promotes metastatic dissemination of otherwise non-aggressive breast cancer cells *in vivo* [36]. miR-30a inhibits EMT by directly targeting Snai1 in non-small cell lung cancer cells [37]. Treatment of mammary epithelial cells with TGFβ induced miR-155, whose knockdown suppressed TGFβ-induced EMT, migration, and invasion through direct targeting of RhoA [38]. miR-204 plays a critical role in maintaining epithelial barrier function and cell physiology by directly targeting TGFβR2 and Snail2 in retinal pigment epithelium [39]. The miR-221/222 miRNA cluster has been found to induce EMT in breast cancer cells [40]. miR-27 is upregulated in gastric cancer metastasis and enhances EMT through regulation of Zeb1, Zeb2 and Slug [41]. These studies support a crucial role of microRNAs in controlling EMT and metastasis.

## EMT IN PROSTATE CANCER

In prostate cancer, EMT has been implicated particularly in the context of metastatic disease [26, 42–44]. E-cadherin expression is commonly lost or reduced in PCa [26, 42]. Loss of E-cadherin and gain of mesenchymal marker, N-cadherin has been associated with multiple end points of progression and cancer related mortality [20–24]. Also, PB-Cre4Ptenfl/flTP53fl/fl mouse model of prostate cancer demonstrate the role of EMT in development of aggressiveness. These mice represent prostatic intraepithelial neoplasia (PIN) at 8 weeks, adenocarcinoma formation by 4 months and the progressive development of EMT and sarcomatoid carcinoma with mesenchymal stem cell characteristics, leading to the death of most animals at about 7 months of age [45, 46]. Also, sarcomatoid carcinomas with a metastatic propensity have been described in humans with prostatic disease [47] supporting the existence of EMT like states in prostate cancer. Benign prostatic hyperplasia (BPH) of the prostate exhibits manifestations of EMT state characterized by accumulation of mesenchymal-like cells derived from the prostatic epithelium and endothelium [48]. TGFβ acts as a tumor suppressor in non-tumorigenic prostate epithelial cells, but promotes EMT in tumorigenic prostate cells [49]. In prostate cancer, TGFβ levels have been reported to increase with tumor burden and PCa metastases, also increasing in circulation [50, 51] and are negatively correlated with prognosis. ZEB1 is also a direct repressor of E cadherin in prostate cancer cell lines, and its level of expression correlates with Gleason score [44,45]. The Wnt signaling pathway has also been implicated in EMT-like states in prostate cancer [26].

## MICRORNAS AND EMT IN PROSTATE CANCER

**Figure 1:**
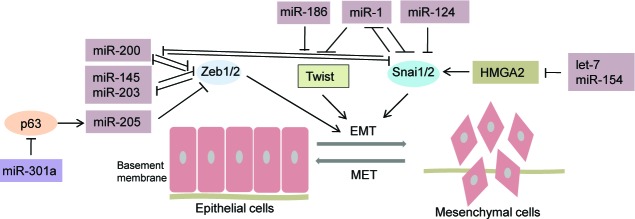
MicroRNAs regulating EMT-TFs in prostate cancer Schematic illustration depicting microRNAs that have been reported to regulate major families of EMT- transcription factors (Zeb, Snail and Twist) in prostate cancer. MicroRNAs mediate PCa EMT by directly repressing single or multiple EMT-TFs. 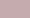 EMT-inhibiting miRNAs, 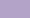 EMT-promoting miRNAs, solid bars denote transcriptional repression, solid arrows denote transcriptional activation. As indicated, microRNAs inhibit EMT by directly or indirectly repressing Zeb1/2, Twist, and/or Snai1/2. miR-200, miR-145, miR-203, miR-205 repress ZEB1/2; miR-1 and miR-186 inhibit TWIST; miR-1 and miR-124 repress SNAI2; miR-301a represses p63 which downregulates miR-205 causing an upregulation of Zeb1/2. In addition, some of the depicted miRNAs participate in double-negative feedback loops with their respective targets as represented for miR-200, miR-145 and miR-1.

**Figure 2:**
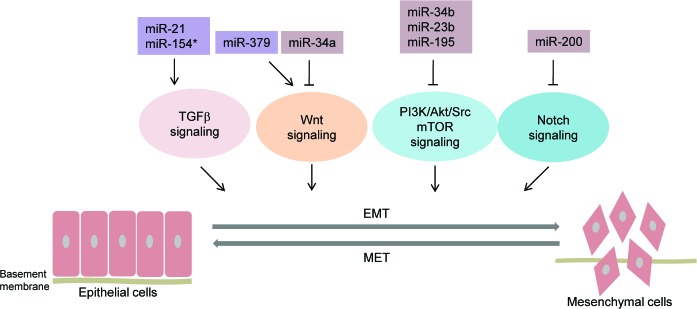
MicroRNAs regulating major signaling pathways involved in EMT in prostate cancer Schematic illustration depicting microRNAs that have been reported to regulate major signaling pathways involved in EMT in prostate cancer. These pathways include TGFβ signaling, Wnt signaling, PI3K/Akt/Src mTOR signaling, and Notch signaling. The key microRNAs regulating these pathways are indicated. 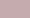 EMT-inhibiting miRNAs, 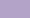 EMT-promoting miRNAs, solid bars denote transcriptional repression, solid arrows denote transcriptional activation. miR-21, miR-154* impinge upon TGFβ pathway; miR-379 and miR-34a promote and inhibit Wnt signaling, respectively. miR-34b, miR-23b, miR-195 inhibit PI3K/Akt/Src/mTOR signaling and miR-200 inhibits Notch signaling.

Studies point to an important role of microRNAs in regulating EMT and MET in prostate cancer, that in turn regulates tumor progression and metastasis (Table 1). MicroRNAs control EMT and MET through efficiently targeting single or multiple components that impinge upon these regulatory pathways via multiple mechanisms- either by (i) directly repressing single or multiple EMT-TFs or regulating cytoskeletal components (epithelial and mesenchymal genes) (Figure 1) or (ii) by regulating key signaling pathways involved in EMT [14, 16, 18]. (Figure 2) Thus, miRNAs impact these pathways in various regulatory modes and their regulation is often controlled by double-negative feedback loops, leading to an efficient and flexible control of EMT. Dysregulation of miRNAs leads to induction of EMT states, leading to tumor progression, recurrence and metastasis. Tumor suppressive miRNAs act by inhibiting EMT and oncogenic microRNAs often act as EMT promoters. The following sections highlight the significant studies on suppressive/oncogenic microRNAs in PCa that act by regulating EMT. An understanding of the role of miRNA-mediated EMT program may help elucidate the mechanistic model of PCa progression and metastasis and identify novel targets for therapeutic intervention.

**Table 1:** MicroRNAs regulating EMT in prostate cancer

MicroRNA	Effects on EMT	Target(s)	References
***miRNAs primarily targeting EMT transcription factors or cytoskeletal components***			
**miR-1**	Inhibit	Slug, Twist1	[55, 58]
**miR-124**	Inhibit	Slug	[70]
**miR-145**	Inhibit	ZEB2, HEF1	[67, 68]
**miR-200**	Inhibit	Zeb1, Zeb2, Slug	[52]
**miR-203**	Inhibit	Zeb2, Bmi1, Survivin CKAP2, LASP1, WASF1, ASAP1 mRNAs	[62, 63]
**miR-205**	Inhibit	Zeb2, Protein Kinase Cε	[60]
**miR-186**	Inhibit	Twist1	[71]
**miR-23a-3p**	Promote	E-cadherin	[105]
***miRNAs targeting signaling pathways implicated in EMT***			
**let 7 family**	Inhibit	HMGA2	[82]
**miR-21**	Promote	BTG2, TGFβ	[106, 110]
**miR-23b**	Inhibit	Src kinase and Akt	[91]
**miR-29b**	Inhibit	MMP2	[95]
**miR-30**	Inhibit	ERG	[92]
**miR-32**	Promote	BTG2	[108]
**miR-34a**	Inhibit	LEF1	[89]
**miR-34b**	Inhibit	Akt	[90]
**miR-100**	Inhibit	AGO2	[93]
**miR-154**	Inhibit	HMGA2	[85]
miR-154*	Promote	SMAD7	[112]
**miR-195**	Inhibit	FGF2, HMGA1, RPS6KB	[97–99]
**miR-223**	Inhibit	ITGA3, ITGB1	[100]
**miR-301a**	Promote	p63	[104]
**miR-331-3p**	Promote	NRP2, NACC1	[114]
**miR-379**	Promote	FOXF2	[112]
**miR-573**	Inhibit	FGFR1	[102]
***miRNAs affecting EMT and PCa stem cells***			
**miR-200**	Inhibit	Notch1	[80]
**Let-7**	Inhibit	EZH2	[80, 125]
**miR-205**	Inhibit	Zeb2, Protein Kinase Cε	[60]
**miR-145**	Inhibit	Zeb2	[67]
**miR-21**	Promote		[124]

Table summarizes the miRNAs that have been reported to inhibit/promote EMT in prostate cancer. Target genes of the respective miRNAs are indicated in column 3.

### MicroRNAs that inhibit EMT

Several tumor suppressive miRNAs act to inhibit PCa EMT by directly repressing one or more EMT-TFs or inhibiting signaling pathway components influencing EMT. These miRNAs negatively regulate tumor progression and metastasis as EMT is considered as the initiating and most critical step in invasion and metastasis [13].

#### EMT-inhibiting miRNAs primarily regulating EMT-TFs

MicroRNAs such as miR-200 family, miR-205 and miR-203 promote the epithelial state and inhibit EMT by primarily repressing EMT-TFs among other targets (Figure 1 and Table 1). Loss of these miRNAs leads to induction of EMT states as highlighted below:

#### miR-200 family and miR-1

The miR-200 family includes miR-200a, miR-200b, miR-200c, miR-141, and miR-429, all of which are significantly downregulated in PCa. Each of these miRNAs has been reported to suppress PCa metastasis via EMT inhibition. miR-200 inhibits Platelet-Derived Growth Factor-D (PDGF-D) induced EMT in PC3 cells by targeting and repressing Zeb1, Zeb2, and Slug expression (Figure 1) [52]. Similarly, miR-200b significantly inhibited tumor growth and cell proliferation in PC-3 cells due to inhibition of EMT [53]. Slabakova *et al* showed that miR-200 can counteract TGFβ1-induced EMT in benign prostate hyperplasia (BPH) cells [54]Liu *et al* identified that miR-200 along with miR-1 act as negative regulators of EMT in *Pten* and *TP53* null murine models [55]. miR-1 and miR-200 repress EMT by targeting Slug, which plays an important regulatory role in mesenchymal differentiation. Further, Slug was found as a direct repressor of miR-1 and miR-200 transcription suggesting that Slug and miR-1miR-200 form a self-reinforcing regulatory loop that leads to EMT amplification (Figure 1) [55]. Also, transcription factor OVOL has been reported to induce miR-200 expression [56] and has been predicted to modulate PCa EMT by interacting with the miR-200/ZEB axis [57]. miR-1 targets Twist1 in PCa [55]. EGFR has been shown to act as a transcriptional repressor of miR-1 leading to oncogenic activation of Twist1, contributing to accelerated PCa bone metastasis [58].

#### miR-205

miR-205 shows significantly lower expression levels in prostate cancer cells in comparison to normal prostate cells [59]. miR-205 overexpression in a prostate cancer cell line led to alterations consistent with MET and a reduction in cell invasion primarily through downregulation of protein kinase Cε [60]. In addition, miR-205 was shown to directly target Zeb2, N-chimaerin, ErbB3, E2F1 and E2F5 in prostate cancer cells [60]. Another study showed that miR-205 is essential for the inhibitory effects of p63 on PCa EMT markers, such as Zeb1, and metastasis (Figure 1) [61]p63 (both TAp63 and ΔNp63 isoforms). ΔNp63 or miR-205 significantly inhibited the incidence of lung metastasis *in vivo* in a mouse tail vein model. Further, in human PCa specimens, partial or complete loss of the ΔNp63/miR-205 axis was found to be correlated with EMT expression patterns and a predictor of metastatic behavior and poor prognosis [61].

#### miR-203

miR-203 is a tumor suppressor miRNA that is downregulated in PCa [62, 63]. We provided first evidence that miR-203 represses EMT by directly targeting Zeb2 (Figure 1) and other EMT regulators, including Bmi1, that in turn regulate invasion and metastasis of prostate cancer [26, 64]. In addition, miR-203 regulates a cohort of pro-metastatic genes including survivin and bone-specific effectors including Runx2, a master regulator of bone metastasis [62, 63]. Upon overexpression in prostate cancer cell lines, miR-203 induces MET and inhibits cell invasion [62, 63]. Viticchie *et al.* demonstrated that miR-203 additionally targets a series of metastasis-promoting genes, including *CKAP2*, *LASP1*, *WASF1* and *ASAP1* mRNAs [63].

#### miR-143/miR-145

miR-143/miR-145 form a cluster and are both reported to be downregulated in metastatic PCa [65]. Peng *et al* identified that downregulation of miR-143 and miR-145 is associated with induction of EMT and PCa bone metastasis [66]. miR-145 is a strong EMT repressor that directly targets Zeb2 [67]. Further, Zeb2 directly targets miR-145, repressing its transcription, thereby forming a double-negative feedback loop that regulates EMT, stemness and PCa bone metastasis (Figure 1). Zeb2 expression was positively correlated with bone metastasis in PCa patients and negatively correlated with miR-145 expression in primary PCa samples [67]. Guo *et al* showed that a cytoplasmic scaffolding protein, human enhancer of filamentation 1 (HEF1; also known as NEDD9/Cas-L), is also a direct miR-145 target. miR-145 and HEF1 expression levels were negatively correlated in primary prostate cancer and bone metastatic specimens [68]. Further, miR-145 expression is controlled by DNA hypermethylation and tumor suppressor p53 in PCa [65, 69]. WT-p53 upregulates the expression of miR-145, thereby suppressing metastasis and EMT in PC3 prostate cancer cells and these effects could be reversed by anti-miR-145. Thus, miR-145 plays at least a partial role in the EMT inhibitory effects of WT-p53 [69].

#### miR-124

Qin et al. found that transforming growth factor-α (TGF-α) promotes EMT through downregulation of miR-124 in prostate cancer cell line. Overexpression of miR-124 on the TGF-α treated PCa cells inhibited the EMT inducing effects of TGF-α and reduced cellular invasion. Further, the authors demonstrated that miR-124 directly targets Slug (Figure 1) [70].

#### miR-186

miR-186 has been reported to be downregulated in human PCa tissues, particularly in metastatic samples and metastatic prostate cancer cell line [71]. Low miR-186 expression was found to be correlated with poor patient survival. miR-186 was found to inhibit EMT and PCa metastasis by directly targeting and downregulating Twist1 [71].

#### *Let7* family

The let-7 family of miRNAs include let-7a, let-7b, let-7c, let-7d, let-7e, let-7f, let-7g, and let-7i [72], all of which are significantly downregulated in PCa, compared to benign tissues and act as tumor suppressors [32]. Lin28 is a highly conserved RNA-binding protein and an embryonic stem cell factor that blocks the processing of let-7 precursors into mature miRNAs [72–74]. Lin28 is frequently overexpressed in primary human tumors [75] and promotes oncogenesis and proliferation of cancer cells, by repression of the let-7 family [76]. Let-7 expression has been reported to be downregulated in localized PCa tissues relative to benign peripheral zone tissue [77, 78]. Loss of let-7 expression has been linked to the development of poorly differentiated and aggressive cancers [79]. The let-7 miRNAs are tumor suppressive and have been shown to target oncogenes such as Ras, Myc and genes involved in cell cycle regulation. These miRNAs thereby target oncogenes at various metastatic steps including EMT [17]. Kong et al. reported an important link between let-7 and miR-200 with EMT states in prostate cancer [52, 80]. One of the let-7 family's target genes in EMT is high mobility group protein A (HMGA), a chromatin associated nonhistone protein [81]

#### miR-154

Similarly, miR-154 inhibits EMT in prostate cancer cells by directly targeting HMGA2 [85]. HMGA2 is mostly upregulated in PCa tissue samples as compared to normal. Overexpression of miR-154 inhibited EMT and reduced migratory and invasive capabilities of PCa cells *in vitro* concomitant with HMGA2 repression (Figure 1) [85].

#### MicroRNAs regulating key signaling pathways implicated in EMT

MicroRNAs also impinge upon key signaling pathways involved in EMT including TGFβ, Wnt, PI3K/Akt and Receptor Tyrosine Kinases (RTKs) to regulate EMT (Figure 2 and Table 1) [18] as summarized below:

#### miR-34a/b

The miR-34 family of tumor suppressive miRNAs include miR-34a/b/c that are encoded by two different genes. miR-34a precursor is transcribed from chromosome 1 while miR-34b and miR-34c precursors are co-transcribed from a region on chromosome 11 [86]. miR-34 is an important component of the p53 network and is directly transactivated by p53 [86, 87]. miR-34 has been reported to target key genes that function in cell cycle, apoptosis, senescence, cell migration, and invasion [86]. In mice, prostate epithelium-specific inactivation of miR-34 and p53 leads to early invasive adenocarcinomas and high-grade prostatic intraepithelial neoplasia with expansion of the prostate stem cell compartment [88]. miR-34a has been reported to be significantly downregulated in human PCa specimens in contrast to benign prostate tissues [89]. Further, it has been demonstrated that miR-34a regulates PCa EMT by directly repressing LEF1 (lymphoid enhancer-binding factor-1), a transcription factor in the Wnt signaling pathway that plays a role in cell proliferation and invasion (Figure 2) [89]. We reported that miR-34b is tumor suppressive in PCa and is silenced through CpG hypermethylation [90]. Reconstitution of miR-34b inhibited cell proliferation/invasion and EMT by directly targeting Akt and its downstream proliferative genes [90].

#### miR-23b

miR-23b is a methylation-silenced tumor suppressor in prostate cancer that works by directly targeting proto oncogene Src kinase and Akt (Figure 2) [91]. Overexpression of miR-23b in prostate cancer cells led to a decrease in mesenchymal markers and an increase in epithelial markers suggesting that miR-23b inhibits EMT and promotes MET [91].

#### miR-30

In PCa, Ets-related gene (ERG) is a frequently overexpressed oncogene that is activated by fusion events between promoter sequences of the TMPRSS2 and coding sequences of ERG. Kao *et al* showed that ERG is a direct target of miR-30 [92]. miR-30 is underexpressed in prostate cancer specimens and its overexpression in prostate cancer cells suppressed EMT and inhibited cell migration and invasion [92]. Further, Src inhibitors upregulated miR-30 levels suggesting that TMPRSS2-ERG gene fusions are regulated by Src via miR-30 [92].

#### miR-100

miR-100 negatively regulated EMT in PC3 and DU145 cells and its expression was negatively correlated with bone metastasis of prostate cancer patients [93]. Downregulation of miR-100 promoted prostate cancer metastasis by upregulating Argonaute 2 (AGO2), a core effector protein of the miRNA-induced silencing complex which facilitates EMT [93].

#### miR-29b

miR-29b is downregulated in PCa cells as compared to normal prostate epithelial cells, and its expression was further lowered in metastatic prostate cancer cells [94]. Upon miR-29b transfection in PC3 prostate cancer cells, E-cadherin was upregulated while N-cadherin, Twist, and Snail expression were downregulated [94]. Owing to its EMT-inhibiting effects, ectopic miR-29b expression suppressed PCa metastasis in a mouse model [94]. Matrix metallopeptidase-2 (MMP-2) has been identified as a miR-29b target in prostate cancer cells [95]. Further, miR-29b has been shown to enhance the chemotherapeutic effects of cisplatin through its direct targeting of DNMT3B (DNA (Cytosine-5-)-Methyltransferase 3 Beta) and Akt3 (v-Akt Murine Thymoma Viral Oncogene Homolog 3) [96].

#### miR-195

miR-195 is downregulated in prostate cancer tissues and low miR-195 is significantly associated with high Gleason score, biochemical recurrence and metastasis [97, 98]. Prostate cancer cases with low miR-195 expression had a shorter relapse-free survival (RFS) time [98]. miR-195 overexpression in DU-145 and PC3 PCa cell lines led to EMT inhibition and reduced invasion and migration by directly targeting fibroblast growth factor 2 (FGF2) [98]. Also, miR-195 has been reported to directly repress HMGA1 in prostate cancer [99]. Another study identified Ribosomal protein S6 kinase, RPS6KB, an important component of mTOR signaling pathway as a novel direct miR-195 target (Figure 2) [97]. The latter study suggests that miR-195 loss upregulates RPS6KB1, leading to a decreased expression of E-cadherin and increased expression of MMP-9 and VEGF proteins that culminates in effects on tumor invasion, angiogenesis, EMT, and survival [97].

#### miR-223

miR-223 is significantly downregulated in PCa tissues and restoration of miR-223 led to inhibited cell proliferation/invasion [100]. miR-223 was shown to directly target integrin alpha 3 (ITGA3) and integrin β1 (ITGβ1) [100]. These integrins are involved in focal adhesion and extracellular matrix (ECM) receptor interactions that play a role in EMT induction in prostate cancer cells [64, 101].

#### miR-573

miR-573 is downregulated in metastatic PCa in comparison to primary PCa [102]. miR-573 overexpression inhibited TGF-β1 induced EMT *in vitro* and lung metastasis *in vivo* by directly targeting the fibroblast growth factor receptor 1 (FGFR1) gene [102]. FGFR1 plays a critical role in prostate tumorigenesis, and miR-573 directly suppresses FGFR1 expression in PCa cells [102, 103]. Further, miR-573 was found to be directly regulated by transcription factor GATA3 suggesting the involvement of GATA3, miR-573 and FGFR1 in controlling PCa EMT [102].

### MicroRNA that promote EMT

In prostate cancer, various oncogenic microRNAs often repress epithelial characteristics or promote EMT as summarized below.

#### EMT-promoting miRNAs regulating EMT-TFs or cytoskeletal components

Oncogenic miRNAs can promote EMT by augmenting the expression of EMT-TFs indirectly as exemplified by miR-301a or by directly targeting cytoskeletal components (epithelial genes).

#### miR-301a

In a recent study, Nam et al. showed that miR-301a induces EMT in prostate cancer cells by directly targeting and inhibiting the tumor suppressor p63. The loss of p63 results in the downregulation of tumor suppressive miR-205, causing upregulation of ZEB1 and ZEB2 (Fig. 1), which in turn, suppresses E-cadherin [104].

#### miR-23a-3p

Wen et al. demonstrated that miR-23a-3p regulates TGF-β-induced EMT in androgen independent prostate cancer (AIPC) cell lines PC3 and Du145 [105]. Treatment of PCa cell lines with TGF-β led to miR-23a-3p upregulation concomitant with E-cadherin downregulation. Further, miR-23a-3p was found to directly repress E-cadherin expression by binding to its 3′UTR. Also, a bioactive coumarin derivative, osthole, suppressed EMT *in vitro* and metastasis *in vivo* by inhibiting miR-23a-3p expression and Snail signaling in AIPC cells [105].

#### EMT-promoting miRNAs regulating signaling pathways implicated in EMT

In addition, oncogenic miRNAs may repress tumor suppressor genes or act by repressing inhibitors of signaling pathways involved in EMT as summarized below (Table 1).

#### miR-21

miR-21 is commonly upregulated in various cancers, including prostate cancer [106]. miR-21 has been reported to be Ras-induced, leading to tumor aggressiveness [107]. Overexpression of miR-21 in prostate cells promotes EMT leading to the transformation of normal prostate cells into prostate cancer cells [108] primarily from repression of B-cell translocation gene 2 (BTG2). B-cell translocation gene 2 (BTG2) is a basal protein that has been implicated in prostate cancer transformation and progression [108] and also possesses anti-proliferative activity [109]. A recent study shows that miR-21 overexpression concomitant with loss of the miR-15/16 cluster leads to aberrant TGFβ signaling, EMT, increased aggressiveness and PCa metastasis [110].

#### miR-32

Jalava *et al* showed that miR-32 also targets BTG2 and that their expression levels are inversely correlated in castration-resistant prostate cancer (CRPC) [111]. miR-32 is an androgen-regulated miRNA that is highly expressed in CPRC samples leading to reduced expression of BTG2 [111].

#### miR-154* and miR-379

miR-154* and miR-379 are members of the delta-like 1 homolog-deiodinase, iodothyroxine (DLK1-DIO3) cluster - a cluster that plays an important role in EMT and embryonic development. Gururajan et al. demonstrated that both miR-154* and miR-379 have elevated expression in bone metastatic PCa cell lines and tissues and miR-379 expression correlated with progression-free survival of PCa [112]. Overexpression of these miRNAs promoted EMT in PCa [112] while their inhibition resulted in EMT reversal and decreased invasive capacities of PCa cells [112]. Analyses of the potential target pathways showed that these miRNAs regulate oncogenic pathways that activate EMT such as E2F signaling, Ras pathway, WNT and TGFβ pathways. [112]. miR-154* targets SMAD7, an inhibitor of the TGFβ pathway [113] while miR-379 was predicted to inhibit forkhead box F2 (FOXF2), a WNT pathway inhibitor (Figure 2) [112].

#### miR-331-3p

miR-331-3p expression promotes EMT as its overexpression in the PC3 cell line led to upregulation of mesenchymal markers such as vimentin and N-cadherin, and downregulation of epithelial markers such as E-cadherin and desmoplakin [114]. Further, the authors demonstrated that miR-331-3p- mediated EMT was due to direct targeting of Neuropilin 2 (NRP2) and Nucleus Accumbens-Associated Protein 1(NACC1) expression.

miR-331 expression is elevated in PCa cases with high Gleason score and was shown to be controlled by Syndecan-1 [114].

#### miR-129-3p

miR-129-3p has been reported to be upregulated in metastatic PCa cells [115]. miR-129-3p was found to directly repress Centriolar Coiled-Coil Protein 110kDa (CP110), a centrosomal protein that is downregulated in metastatic PCa. Lack of CP110 inhibition led to excessive centrosome amplification accompanied by increased E-cadherin expression, deregulated F-actin cytoskeleton and decreased invasion and metastasis [115]. Though a direct correlation was observed between CP110 expression, E-cadherin expression and PCa metastases, no significant correlation could be established between CP110 expression and the expression of EMT-related genes in clinical prostate cancer samples [115].

#### EMT promoting miRNAs with undefined mechanisms miR-409

miR-409-3p/-5p expression is elevated in bone metastatic PCa cell lines and in PCa tissues with higher Gleason scores [116]. High miR-409-3p expression was found to be correlated with progression-free survival of PCa patients. Further, orthotopic delivery of miR-409-3p/-5p in the murine prostate gland induced EMT and tumors expressing EMT and stemness markers. Inhibition of miR-409-5p in the bone metastatic ARCaPM prostate cancer cell line led to decreased bone metastasis and increased survival in an experimental mouse model [116].

### MicroRNAs, EMT and prostate cancer stem cells

Prostate cancer recurrence and progression has been associated with the existence of prostate cancer stem cells (CSC) or tumor-initiating cells (TIC). These cells are refractory to current therapies [117] and are characterized by various cell surface markers [118–122] such as CD44, CD133, integrin α2β1 and stem-cell signature genes such as Sox2, Nanog and Oct4. EMT endows cancer cells with stem cell properties [19, 123]. It has been reported that EMT is mechanistically linked to stem cell signatures in prostate cancer [80]. Several miRNAs implicated in EMT have been reported to influence prostate cancer stem cells indirectly by regulating EMT-TFs or by influencing the signaling pathways that impinge upon both EMT and stemness such as TGFβ, MAP kinase and Wnt signaling pathways [19, 123]. Decreased expression of miR-200 and/or let-7 family has been reported in PCa cells that have undergone EMT and display stem cell- like features [80]. miR-200 reexpression led to reversal of EMT and inhibition of stem cell properties such as decreased prostasphere-forming ability and clonogenicity concomitant with reduced expression of Notch1 and Lin28B [80]. Similarly, miR-205 reverses EMT progression and inhibits stem-cell properties of PCa cells through repression of its targets Zeb2 and protein kinase Cε [60]. miR-145 inhibits EMT and stem cell properties in PCa [67] via its regulation of Zeb2. miR-21 is involved in EMT, maintenance of CSCs and therapeutic resistance [124]. Downregulation of miR-21 led to repression of EpCAM, CD44 and VEGF [124]. Let-7 directly targets Enhancer of Zeste homolog 2 (EZH2) and thereby regulates PCa CSCs. Loss of let-7 during PCa progression leads to increased expression of EZH2, contributing to PCa aggressiveness [125]. In addition to influencing CSCs indirectly through EMT, miRNAs can also regulate CSCs via their ability to directly regulate CSC markers [123]. As an example, miR-34a directly represses CD44 thereby inhibiting PCa stem cells and metastasis [126–128]. We showed that miR-708 is a key negative regulator of the CD44(+) subpopulation of prostate cancer cells by directly regulating CD44 [129]. Also, miR-708 directly targets Akt2, a component of the PI3K/Akt pathway, with roles in tumor progression and CSCs [129].

## CONCLUSIONS AND FUTURE PERSPECTIVES

In conclusion, the role of miRNAs in PCa EMT is emerging and warrants further investigation. Studies till date suggest that microRNAs mediate efficient and reversible post-transcriptional control of prostate cancer EMT via multiple mechanisms including direct targeting of EMT-TFs, affecting signaling pathway components controlling EMT or by directly modulating cytoskeletal components. Oncogenic microRNAs often act as EMT promoters by repressing epithelial characteristics and tumor suppressive miRNAs act by inhibiting mesenchymal progression. Loss of EMT-inhibiting miRNAs and/or gain of EMT promoting miRNAs lead to induction of PCa EMT, leading to tumor progression, metastasis and recurrence. Restoring expression of tumor suppressive miRNAs and inhibiting oncogenic miRNAs represent therapeutic opportunities to prevent PCa metastasis and recurrence.
